# Synaptic Plasticity Modulation of Neuromorphic Transistors through Phosphorus Concentration in Phosphosilicate Glass Electrolyte Gate

**DOI:** 10.3390/nano14020203

**Published:** 2024-01-16

**Authors:** Dong-Gyun Mah, Hamin Park, Won-Ju Cho

**Affiliations:** 1 Department of Electronic Materials Engineering, Kwangwoon University, Gwangun-ro 20, Nowon-gu, Seoul 01897, Republic of Korea; 2 Department of Electronic Engineering, Kwangwoon University, Gwangun-ro 20, Nowon-gu, Seoul 01897, Republic of Korea

**Keywords:** phosphosilicate glass, electrolyte gate, synaptic transistor, phosphorus concentration, electric double layer, neuromorphic system

## Abstract

This study proposes a phosphosilicate glass (PSG)-based electrolyte gate synaptic transistor with varying phosphorus (P) concentrations. A metal oxide semiconductor capacitor structure device was employed to measure the frequency-dependent (C-*f*) capacitance curve, demonstrating that the PSG electric double-layer capacitance increased at 10^3^ Hz with rising P concentration. Fourier transform infrared spectroscopy spectra analysis facilitated a theoretical understanding of the C-*f* curve results, examining peak differences in the P-OH structure based on P concentration. Using the proposed synaptic transistors with different P concentrations, changes in the hysteresis window were investigated by measuring the double-sweep transfer curves. Subsequently, alterations in proton movement within the PSG and charge characteristics at the channel/PSG electrolyte interface were observed through excitatory post-synaptic currents, paired-pulse facilitation, signal-filtering functions, resting current levels, and potentiation and depression characteristics. Finally, we demonstrated the proposed neuromorphic system’s feasibility based on P concentration using the Modified National Institute of Standards and Technology learning simulations. The study findings suggest that, by adjusting the PSG film’s P concentration for the same electrical stimulus, it is possible to selectively mimic the synaptic signal strength of human synapses. Therefore, this approach can positively contribute to the implementation of various neuromorphic systems.

## 1. Introduction

The rapid evolution of artificial intelligence (AI) technology in contemporary society has revolutionized problem solving across diverse fields, including healthcare, education, transportation, the environment, and the economy [1,2,3,4,5]. By efficiently processing vast amounts of data according to specific needs, AI plays a pivotal role in enhancing quality of life for humans. Despite the widespread use of the von Neumann architecture for its ability to store and process large datasets, it faces challenges such as the imbalance between advancements in storage and processing speeds, inefficient energy consumption for data transfer, and difficulties in handling real-time issues like autonomous driving. These challenges arise from the von Neumann architecture’s reliance on a serial approach with separate storage and processing units [6,7,8,9]. These challenges indicate a pressing need for a new architecture, prompting active research to develop computing systems that mimic the network of the biological nervous system [10]. The key is to create systems that operate with low power (~20 W) and which rapidly store and parallelly distribute vast amounts of data. The human brain, with approximately 10^12^ neurons and 10^15^ synapses, serves as an inspiration. Each neuron is connected to around 10^3^ different neurons, and synapses are crucial in connecting two neurons and transmitting electrical signals between them [11,12,13]. Synaptic strength varies with the amplitude of the transmitted signal, and this strength can be controlled through a learning process involving synaptic plasticity. Hardware implementation of neuromorphic computing systems is essential in mimicking the strength of electrical signals in synapses [14,15]. Many research groups are exploring various types of synaptic transistors, including organic- and inorganic-based ferroelectric gate synaptic transistors, electrolyte gate synaptic transistors, and optoelectronic synaptic transistors. Electrolyte gate synaptic transistors utilize cation or anion movements in the electrolyte to modulate channel conductivity. The time required for charging and discharging ion capacity is very similar to the time scale of biological synapses [16,17,18].

In this study, we focused on controlling the internal phosphorus (P) concentration of phosphosilicate glass (PSG)-based electrolyte-gated transistors (EGTs) and analyzed the associated synaptic characteristics [19,20,21]. The electrolyte film was efficiently produced using a spin-coating method, facilitating the systematic analysis of synaptic characteristics at various P concentrations [22]. Initially, PSG-based metal oxide semiconductor (MOS) capacitors were fabricated with varying P concentrations, and the frequency-dependent (C-*f*) capacitance curves were examined [23]. Interestingly, an upward trend in the PSG electric double-layer (EDL) capacitance was observed in the low-frequency range as the P concentration increased. Chemical structure analysis based on the P concentration within the electrolyte film enabled a comprehensive understanding of the frequency-dependent curves [24,25]. Subsequently, indium gallium zinc oxide (IGZO) synaptic transistors with a PSG-EDL gate dielectric (PSG-EDLT) were fabricated, and differences in the hysteresis window were observed through double-sweep transfer curves based on the P concentration [26,27]. To analyze the characteristics of synaptic transmission strength based on the P concentration, we measured basic artificial synaptic behaviors such as excitatory post-synaptic currents (EPSCs), paired-pulse facilitation (PPF), signal-filtering functions, resting current levels based on the number of pre-synaptic spikes, and potentiation and depression characteristics for three types of EDLT with different P concentrations. Furthermore, we demonstrated the applicability of the proposed synaptic transistor in neuromorphic systems through the Modified National Institute of Standards and Technology (MNIST) learning simulations. The measurement of artificial synaptic behaviors confirmed that PSG-EDLT, depending on the P concentration, could selectively mimic synaptic strength for the same stimulus.

Furthermore, especially in the field of neuromorphic computing systems, the complex dynamics of environmental noise, random fluctuations, and intricate interactions within complex systems have been prominently emphasized. Recent developments, inspired by Giorgio Parisi’s Nobel Prize in Physics, have underscored the significant role of these elements. In this context, the application of various synaptic devices based on phosphorus materials is expected to offer a positive prospect by addressing complex issues related to synaptic operations under conditions of environmental noise, providing new perspectives for applications in neuromorphic computing [28,29,30,31,32,33,34,35].

## 2. Materials and Methods

### 2.1. Material Specifications

The materials used in this study include 20B spin-on glass (Filmtronics Inc., Butler, PA, USA), P509 spin-on dopant (Filmtronics Inc., USA), IGZO sputter target (In_2_O_3_:Ga_2_O_3_:ZnO = 4:2:4.1 mol%, THIFINE Co., Ltd., Incheon, Republic of Korea), and Al pellet (purity > 99.999%; THIFINE Corp., Incheon, Republic of Korea), along with a p-type Si substrate (plane, (100); resistivity range, (1 to 10) Ω·cm; LG Siltron Inc., Gumi, Republic of Korea).

### 2.2. Synthesis of PSG Electrolyte Films

To create the PSG electrolyte film with various phosphorus concentrations, a blend of a spin-on glass (SOG) solution and a spin-on dopant (SOD) solution was utilized. The mass ratios of SOG to SOD ranged from 0:10 to 9:1 in increments of 1, leading to a total of 10 cases. We determined the phosphorus concentration in the blended solution of SOG and SOD based on the following Formula (1):


(1) $$P_{SOD+SOG}=m_{SOD}\times P_{SOD}/(m_{SOD}{+m}_{SOG}),$$

Here, P_SOD+SOG_ represents the phosphorus concentration in the blended solution, P_SOD_ is the phosphorus concentration in the SOD solution, m_SOD_ and m_SOG_ are the mass of the SOD and the SOG solution, respectively. SOG consists of a polysilicate polymer, ethanol (C_2_H_6_O), 2-propanol (C_3_H_8_O), acetone (CH_3_COCH_3_), and water (H_2_O). Heat treatment of SOG at high temperatures removes impurities and solvents, insulating it like SiO_2_ based on the Si-O structure [36]. SOD comprises ethanol (C_2_H_6_O), water (H_2_O), and phosphorus silicate polymer, with a 15% internal phosphorus mass ratio. High-temperature heat treatment removes impurities, forming P-O and P-OH groups, resulting in P-doped SiO_2_ [37]. The solutions, stored below −2 degrees, were mixed in this experiment. The solutions were stirred at room temperature using a magnetic stirrer for 8 h to prevent particle formation and ensure a uniform film on the substrate during spin-coating. This prevented the formation of a thicker film and potential cloudiness from condensation during spin-coating with a cold solution [38].

### 2.3. Fabrication of PSG Electrolyte-Based EDLT

EGTs based on varying levels of P-doped PSG thin films were fabricated in this study. P-type Si substrates used as gates were cleaned using the wet-chemistry-based standard Radio Corporation of America (TCA) cleaning process. The blended SOG and SOD solution was spin-coated onto a 1 × 1 cm^2^ p-Si substrate at 6000 rpm for 30 s. Subsequently, pre-baking was conducted on a hot plate with the following temperature profile: 70 °C for 3 min, 100 °C for 2 min, 150 °C for 2 min, and 200 °C for 2 min. Further, the blended solution electrolyte film was cured, and impurities were removed by thermal annealing performed at 650 °C for 1 h in a forming gas (5% H_2_ + 95% N_2_) to prepare a ~300 nm thick PSG electrolyte film. Subsequently, a 50 nm thick IGZO channel layer with dimensions (width × length) of 120 µm × 60 µm was deposited using RF magnetron sputtering. Finally, a 150 nm thick Al film was deposited by an E-beam and lifted off to form source/drain (S/D) electrodes with dimensions (width × length) of 150 μm × 120 μm.

### 2.4. Method of Characterizations

The fabricated PSG-EDLTs were stored to prevent exposure to external light and humidity. Considering the humidity, which has a significant impact on the PSG-EDL mechanism, we conducted measurements while maintaining relative humidity between 40% and 50%. Fourier transform infrared spectroscopy (FT-IR) using a Bruker Optics ALPHA instrument was employed to discern the molecular structure of the PSG films at varying P concentrations. The capacitance versus frequency (C-*f*) characteristic curves of Al/PSG-EDL/p-Si MOS capacitors were analyzed using the Agilent 4284A precision LCR meter (Hewlett-Packard Corp., Palo Alto, CA, USA). The electrical properties and synaptic functions of the P-doped PSG-EDLTs were characterized using an Agilent 4156B precision semiconductor parameter analyzer (Hewlett-Packard Corp., USA). Additionally, two identical Agilent 8110A pulse generators (Hewlett-Packard Corp., USA) were used to apply electrical pre-synaptic and post-synaptic stimulation to validate the synaptic behavior of the fabricated EDLTs.

## 3. Results and Discussion

### 3.1. Electrical Characteristics of PSG-Based MOS Capacitors

**Figure 1:**
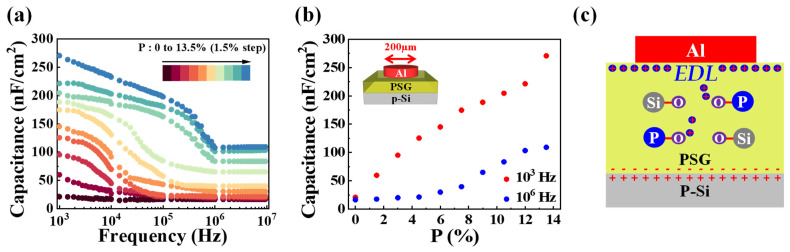
(**a**) Capacitance–frequency (C-*f*) curves of Al/PSG-EDL/p-Si MOS capacitors. (**b**) Capacitance values at 10^3^ and 10^6^ Hz of PSG electrolyte films as a function of P concentration. (**c**) Mechanism of the mobile proton in PSG-EDL.

We fabricated MOS capacitor devices with Al (a diameter of 200 µm)/PSG-EDL/p-Si structures to elucidate synaptic characteristics. The P concentrations ranged from 0% to 13.5% in 1.5% increments, forming ten distinct PSG-EDLs. In Figure 1a, the frequency-dependent (C-*f*) capacitance curves, measured across the frequency range of 10^3^ to 10^6^ Hz, reveal an intriguing trend. The PSG-EDL capacitance notably increases with the rise in P concentration [39]. Figure 1b organizes data according to P concentration, presenting capacitance values at 10^3^ and 10^6^ Hz. For devices with P concentrations ranging from 0% to 4.5%, the capacitance at 10^6^ Hz consistently displays small values, approximately 20 nF/cm^2^, regardless of frequency. However, starting from P 6%, the capacitance shows a substantial increase with the ascending P concentration, reaching a peak of 109.54 nF/cm^2^. At 10^3^ Hz, a discernible trend is observed across P concentrations from 0% to 13.5%, with capacitance escalating from 20 nF/cm^2^ to 271.13 nF/cm^2^. FT-IR spectra analysis was conducted on three types of PSG electrolyte films with varying P concentrations (1.5%, 6%, and 10.5%) to gain insights into the capacitance characteristics at different frequencies among the ten devices. Figure 1c illustrates the physical mechanism of the PSG-EDL in the proposed device. Hydroxyl groups induce the formation of protons within the electrolyte film, and the generated protons accumulate at the channel–PSG interface through a sequence of hopping. Additionally, the influence of phosphorus leads to a reduction in O-H bonding strength, promoting the formation of protons within the PSG and enhancing their mobility.

### 3.2. EDL Operation of PSG Films for Synaptics

**Figure 2:**
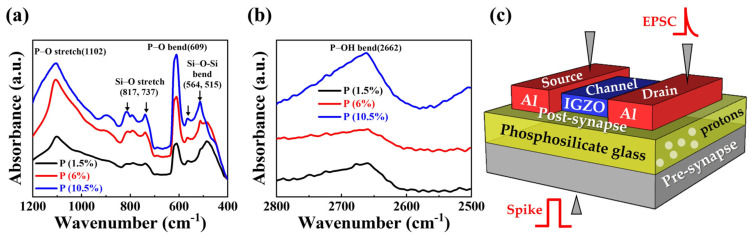
FT-IR spectra according to phosphorus concentration of PSG electrolyte film: (**a**) 1200–400 cm^−1^ and (**b**) 2800–2500 cm^−1^. (**c**) Schematic of the proposed PSG-EDLT.

In Figure 2a, the 1200 cm^−1^ to 400 cm^−1^ range results reveal the presence of P-O bend and P-O stretch at 1102 cm^−1^ and 609 cm^−1^, respectively. Additionally, Si-O-Si bend and Si-O stretch were observed at 564, 515 cm^−1^, and 817, 737 cm^−1^, respectively [40,41,42]. Although each peak intensity varies depending on the P concentration, FT-IR analysis verified that the three types of PSG films are based on the composition of the silicon oxide film [43]. In Figure 2b, the 2500 cm^−1^ to 2800 cm^−1^ range results indicate the observation of the P-OH bend at 2662 cm^−1^. Notably, the intensity of the P-OH peak increases with the corresponding rise in P concentration [44]. The P-OH structure typically generates proton (H^+^) cations, facilitating their movement to the electrode and channel [45,46,47]. Therefore, recognizing that the P-OH structure is the core of capacitance capacity changes with P concentration, we fabricated PSG-EDL gate insulator transistors representing the characteristics of P-doped SiO_2_ for three types of PSG electrolyte films. In Figure 2c, a schematic diagram of the PSG-EDLT is presented. The p-Si substrate serves as the gate, and an IGZO channel with source/drain Al electrodes is deposited on top of the PSG film [48].

**Figure 3:**
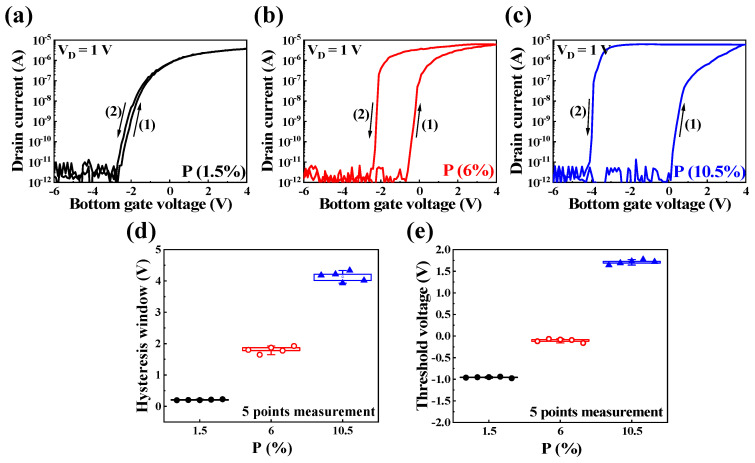
Double-sweep transfer curves at a constant drain voltage (V_D_) of 1 V, with gate voltage ranging from −6 V to 4 V, for various P concentrations: (**a**) 1.5%, (**b**) 6%, and (**c**) 10.5%. (**d**) Hysteresis window and (**e**) threshold voltage corresponding to the double-sweep transfer curves (P concentrations = 1.5%, 6%, and 10.5%).

Figure 3a–c show the double-sweep transfer curves (I_D_-V_G_) of PSG-EDLTs with P concentrations of 1.5%, 6%, and 10.5%, respectively. The gate voltage (V_G_) ranges from −6 V to 4 V, while the drain voltage (V_D_) is set at 1 V. The transfer curve displays counterclockwise hysteresis, transitioning from the forward sweep (1) to the backward sweep (2) as the bottom gate voltage (V_G_) switches. Notably, increased P concentration causes a larger hysteresis window, attributed to the enhanced movement of proton (H^+^) cations facilitated by the P-OH structure. Additionally, the threshold voltages for all three devices exhibit variations.

Figure 3d and Figure 3e represent the hysteresis window and threshold voltage for the three types of devices in the double-sweep transfer curves, respectively. Each device was measured at five different points to ensure the reliability of the results. Upon calculating the average values, the hysteresis window increased from 0.2 V to 4.12 V, and the threshold voltage changed from −0.98 V to 1.79 V. The threshold voltage (V_th_) was determined through extrapolating within the linear region for the transfer characteristics. Ultimately, discernible trends in hysteresis values and threshold voltage corresponding to P concentration were observed. Based on the transfer curve results of PSG-EDLTs with P concentrations of 1.5%, 6%, and 10.5%, we conducted an in-depth analysis and comparison of the synaptic characteristics of the three types as synaptic transistors.

### 3.3. Synaptic Characteristics of P-Doped PSG-Based EDL Synaptic Transistors

**Figure 4:**
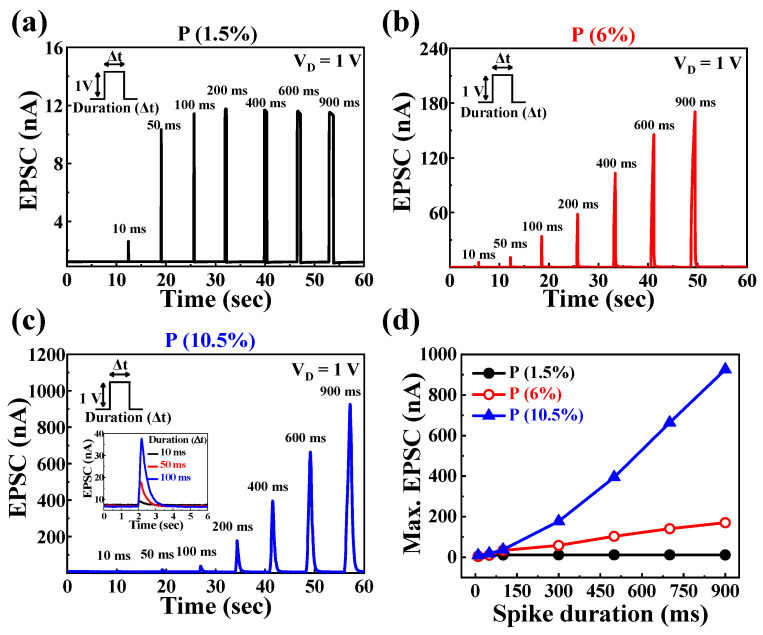
EPSC generated by a single pre-synaptic spike with a fixed amplitude of 1 V over various durations (10 ms to 900 ms) as a function of (**a**) P 1.5%, (**b**) P 6%, and (**c**) P 10.5% concentrations. The inset in (**c**) denotes the P 10.5% EPSCs for durations of 10, 50, and 100 ms. (**d**) Maximum EPSC values from 10 ms to 900 ms.

EPSCs are a fundamental indicator of synaptic weight changes in response to electrical stimulation, modulated by controlling ion flow in the EDLT [49]. In the P-doped PSG-EDLT configuration, the p-Si bottom gate acts as the pre-synapse, the IGZO channel functions as the post-synapse, and both the proton concentration and mobility in the P-doped PSG electrolyte film is crucial for signal transmission. In this study, identical pulses (pre-synapse) were applied to each p-Si bottom gate, and the characteristics of proton mobility within the P-doped PSG electrolyte, depending on the P concentration, were visually distinguished through the IGZO channel (post-synapse). The current generated in the IGZO channel represents the EPSC. With the pre-synaptic spike amplitude fixed at 1 V, the single pulse duration was divided into seven regions, ranging from 10 ms to 900 ms [50]. The corresponding EPSC values of EDLT with P concentrations of 1.5%, 6%, and 10.5% were verified through Figure 4a, Figure 4b, and Figure 4c, respectively. For P 1.5%, with a pre-synaptic spike duration of 10 ms, the maximum EPSC value increased to 2.6 nA, reaching 10.39 nA at 50 ms. However, from 100 ms onward, the maximum EPSC value remained below 11.77 nA, with little variation. This is attributed to the small hysteresis window value of 0.2 V in the double-sweep transfer curve with a bottom gate voltage of −6 V to 4 V. For the P 6% device, as the spike duration increases from 10 ms to 900 ms, the maximum EPSC value increases from 6.69 nA to approximately 170.48 nA, showing an increase of over 25 times with the duration change. This is attributed to the simultaneous influence of increasing proton concentration and mobility in the PSG electrolyte with the rise in P concentration, leading to enhanced channel conductivity and concentration gradient at the PSG electrolyte/channel interface. For the P 10.5% device, the maximum EPSC value increased from 10.46 nA to approximately 929.78 nA, showing an increase of over 88 times, indicating the most prominent EDL characteristics. Figure 4d organizes the data by P concentration for the maximum EPSC values based on the duration of a single spike. For short durations, such as 10 ms, substantial differences in the maximum EPSC values according to P concentration were not observed. Notably, as the spike duration increased for P 6% and 10.5%, a significant variation in the maximum EPSC values was observed. This indicates an increased intensity of signal transmission with longer pre-synaptic single spikes.

**Figure 5:**
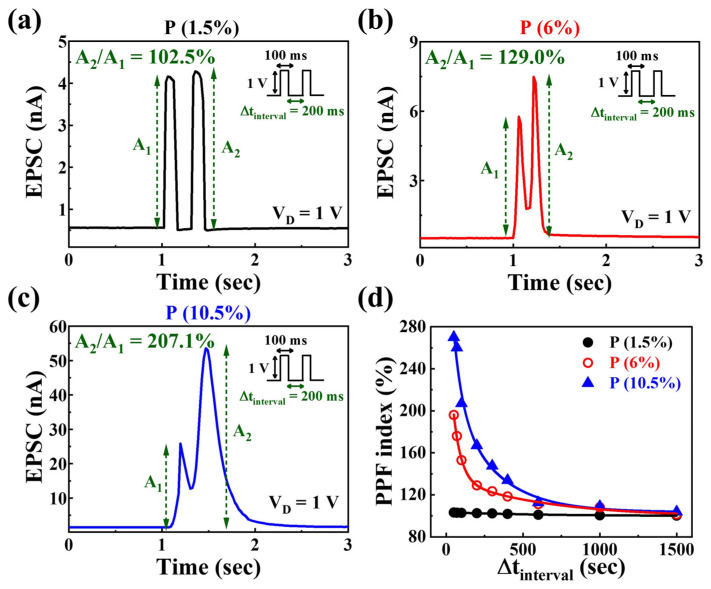
Paired-pulse facilitated EPSC by a paired pre-synaptic spike (1 V, 100 ms) at ∆_tinterval_ = 200 ms as a function of (**a**) P 1.5%, (**b**) P 6%, and (**c**) P 10.5% concentrations. (**d**) PPF index (A_2_/A_1_) plotted against various spike time intervals from 50 ms to 1500 ms.

PPF signifies short-term synaptic plasticity in the nervous system and is pivotal for interpreting biological temporal information, such as auditory or visual data. PPF is contingent on the synaptic spike time interval, where the synaptic potential or current hinges on the time gap (∆t_interval_) between two pre-synaptic spikes. When the second pre-synaptic spike is delivered at the interval of ∆t_interval_ with the same amplitude as the first spike, the post-synaptic potential or current induced by the second spike is amplified [51]. Figure 5a–c illustrate the EPSCs induced by two identical pre-synaptic spikes with a spike ∆t_interval_ of 200 ms for PSG electrolyte films at different P concentrations. For intervals shorter than the complete relaxation time of charge carriers, the characteristics of the relaxation time of charge carriers are clearly distinguished concerning P concentration. It can be observed that, as the P concentration increases, the complete relaxation time of charge carriers becomes longer. Protons induced by the incompletely relaxed first spike contribute to the protons induced by the second spike, increasing the channel current.

Figure 5d represents the fitting data showing the PPF index according to P concentration. PPF can be calculated as the ratio of the first EPSC (A_1_) to the second EPSC (A_2_). The obtained PPF index can be fitted using the following double–exponential decay relationship [52]:

(2) $$PPF\;index={A+C}_{1}\exp(-\Delta t/\tau_{1})+C_{2}\exp(-\Delta t/\tau_{2})$$

where C_1_ and C_2_ denote the initial facilitation magnitudes, while τ_1_ and τ_2_ indicate the characteristic relaxation times. The values of τ_1_ and τ_2_ for the three devices are 9520 ms and 9704 ms, 46.4 ms and 542.4 ms, and 269.9 ms and 329.9 ms, respectively. These results are based on the strengthening of the EDL retention characteristics, where the greater accumulation of protons in the EDL, especially after the first pulse, signifies enhanced EDL retention properties. Moreover, as the EDL retention characteristics become stronger during subsequent pulses, it indicates an increase in synaptic weight effects. As the P concentration increases, these values exhibit characteristics similar to those of typical biological synapses [53,54].

**Figure 6:**
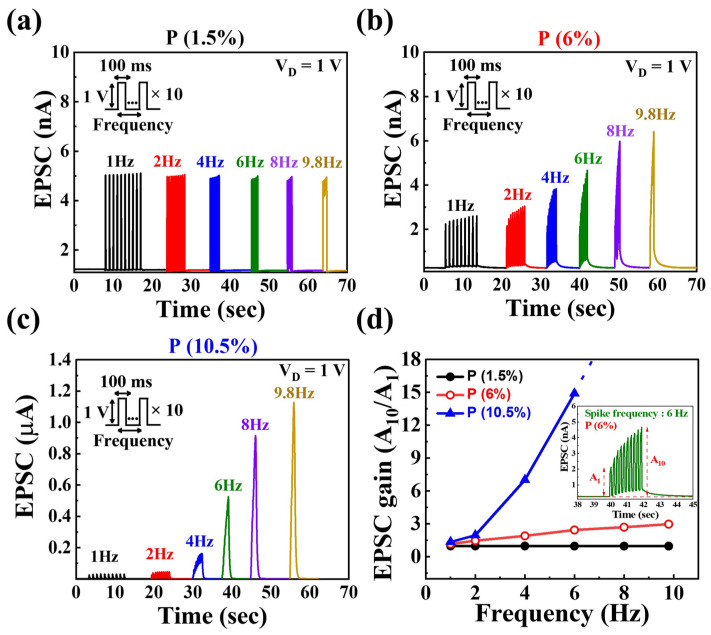
EPSC responses generated by sequential pre-synaptic spikes (1 V, 100 ms) with various frequencies from 1 Hz to 9.8 Hz for (**a**) P 1.5%, (**b**) P 6%, and (**c**) P 10.5% concentrations. (**d**) Pre-synaptic spike-frequency-dependent EPSC gains (A_10_/A_1_). Inset denotes the EPSC response to 6 Hz for P 6% concentration.

Additionally, short-term synaptic plasticity can facilitate the use of dynamic filters for information transmission based on signal frequency. In other words, short-term synaptic facilitation and depression contribute to high-frequency and low-frequency temporal filtering, respectively [55,56]. Figure 6a–c illustrate the EPSC responses to ten consecutive pre-synaptic spikes with varying frequencies from 1 Hz to 9.8 Hz (amplitude 1 V, duration 100 ms). At a frequency of 1 Hz, there was no significant difference between the first spike EPSC values and the 10th spike EPSC values for all three types of devices. Despite the increase in frequency, the P 1.5% device exhibited a similar trend as the 1 Hz case. However, increased P concentration showed a notable rise in the maximum EPSC value with increasing frequency. Figure 6d depicts the EPSC gain as a function of pre-synaptic spike frequency, calculated as the ratio between the first EPSC (A_1_) and the tenth (A_10_). For P 1.5%, despite the frequency increase from 1 Hz to 9.8 Hz, the EPSC gain remained around 1, indicating unsuitability for short-term synaptic facilitation. In the case of P 6%, the EPSC gain increased from 1.16 to 2.9, while for P 10.5%, it was observed that the EPSC gain values exponentially increased, especially at 8 Hz and 9.8 Hz, limiting the distinction between consecutive spikes. Therefore, the proposed synaptic transistor successfully demonstrated the operation of a high-pass filter for information processing with controlled P concentration [57].

**Figure 7:**
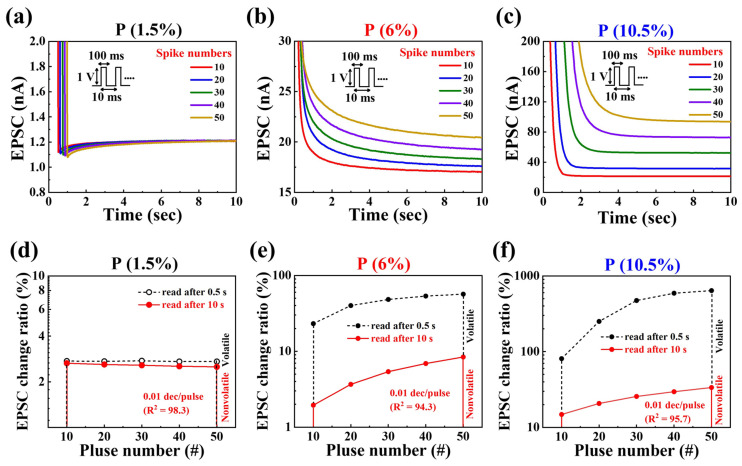
Post-synaptic EPSC response to varying numbers of pre-synaptic stimulus spikes (1 V, 100 ms) for different P concentrations: (**a**) P 1.5%, (**b**) P 6%, and (**c**) P 10.5%. Dependence of the EPSC change ratio ((I − I_0_)/I_0_ × 100%), according to the pulse spike numbers for (**d**) P 1.5%, (**e**) P 6%, and (**f**) P 10.5% concentrations. I_0_ is the resting current, and I is the EPSC in the channel after the pre-synaptic stimulus.

Figure 7a–c show the residual region of the EPSC response according to the number of pre-synaptic spikes (10 to 50 times) for the three devices. Sequential pre-synaptic spikes with an amplitude of 1 V and a duration of 100 ms were applied to the gates at intervals of 10 ms. A concentration gradient generates stimulation, inducing the migration of mobile ions. From the peak value of EPSC following the stimulus, it gradually decreases to various resting current (steady state) levels over milliseconds to 10 s, depending on the P concentration and the number of pre-synaptic spikes. In the case of P 1.5% (Figure 7a), the resting current values remained around 1.2 nA, showing minimal variation with the number of spikes. After the final pre-synaptic stimulus, the EPSC values rapidly decreased over time, indicating unfavorable characteristics for both short-term potentiation (STP) and long-term potentiation (LTP). In contrast, P 6% and P 10.5% show changes in resting current levels from milliseconds to 10 s, depending on the spike numbers (Figure 7b,c). Additionally, it was observed that the slope of EPSC reduction decreased, aligning more closely with the characteristics of synaptic components. This signifies that, with the increase in P concentration, there is an increase in the mobility of ions within the PSG layer, and the number of ions captured between the channel and PSG layer also increases. Particularly, the P 10.5% PSG-EDLT exhibited the most favorable characteristics, with active ion charging and discharging processes, demonstrating superior properties for both STP and LTP.

Figure 7d–f illustrate the pulse-number dependence of the EPSC modulation ratio ((I − I_0_)/I_0_ × 100%), where I_0_ is the resting current and I is the EPSC of the channel after the pre-synaptic stimulus. For P 1.5%, the EPSC change rate shows minimal differences in the volatile and non-volatile regions, with little variation across pulse numbers. However, the EPSC change ratio for P 6% and 10.5% increases with pulse numbers in both the volatile and non-volatile regions. In the non-volatile region, the EPSC change ratio is highest at 10.5%, and the slope of the EPSC change ratio, concerning pulse number, is 0.01 dec/pulse number (R^2^ = 94.3 (P 6%), 95.7 (P 10.5%)). For the post-synaptic residual region following the stimulation by the pre-synaptic pulse, the better the preservation capacity of the EDL, the lower the characteristics of charge neutralization and recombination. This implies that the increase in the non-volatile region with respect to pulse number has a more linear relationship for the P 10.5% device. Therefore, adjusting the P concentration suggests a positive possibility for controlling the STP and LTP strengths in practical applications, implying its potential use in both short-term and long-term memory [58,59].

**Figure 8:**
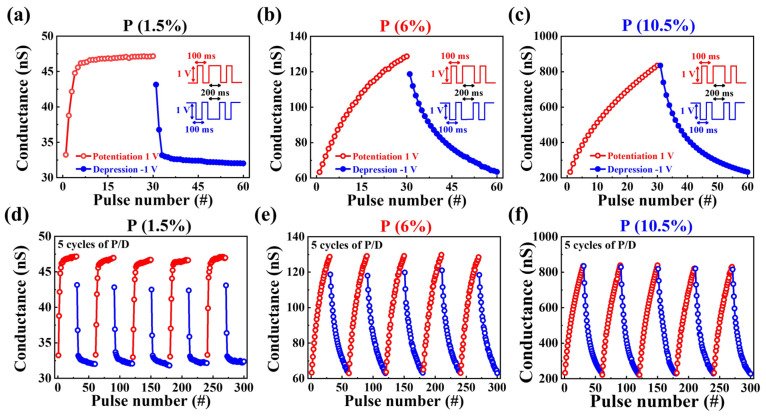
Synaptic weight potentiation and depression characteristics by applying pre-synaptic pulses at (**a**) P 1.5%, (**b**) P 6%, and (**c**) P 10.5% concentrations. Endurance properties over five cycles of potentiation and depression at (**d**) P 1.5%, (**e**) P 6%, and (**f**) P 10.5% concentrations.

Figure 8a–c illustrate the consecutive modulation of channel conductivity in response to repetitive pre-synaptic stimuli, applying 1 V potentiation pulses and −1 V depression pulses for 30 cycles to the three devices, each pulse lasting 100 ms. A read pulse of 1 V was applied to the drain for 200 ms to assess channel conductivity changes. The conductivity exhibits increases and decreases corresponding to the potentiation and depression pulses. For P 1.5%, the values range from 33.27 nS to 47.23 nS for potentiation and from 43.12 nS to 31.97 nS for depression. P 6% shows variations from 63.38 nS to 128.81 nS for potentiation and from 118.89 nS to 63.61 nS for depression. P 10.5% exhibits changes from 232.37 nS to 835.38 nS for potentiation and from 833.53 nS to 233.76 nS for depression. Figure 8d–f depicts the durability characteristics for potentiation and depression over five cycles. All three devices maintained consistent conductance modulation. The conductance values during potentiation remained relatively stable for P 1.5%, ranging from 32.98 nS to 47.23 nS, for P 6% from 62.99 nS to 129.8 nS, and for P 10.5% from 225.95 nS to 838.84 nS. Similarly, during a depression, stable conductance values were observed: P 1.5% ranging from 43.15 nS to 31.81 nS, P 6% ranging from 119.79 nS to 63.05 nS, and P 10.5% ranging from 834.84 nS to 229.42 nS. The consistent modulation of conductance through multiple cycles underscores the robustness of the proposed PSG-based synaptic transistor in facilitating controlled and reliable channel conductivity changes in response to repetitive synaptic stimuli. These findings further highlight the device’s potential for applications requiring sustained and reproducible modulation of synaptic strength.

### 3.4. MNIST ANN Simulation of Proposed Synaptic Transistors

**Figure 9:**
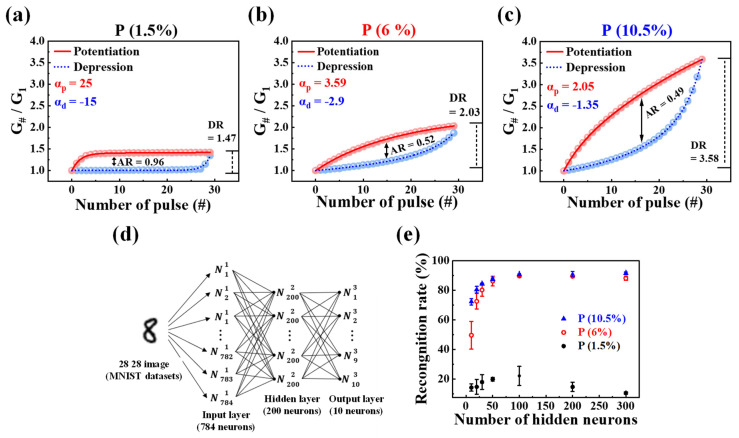
Nonlinearity analysis of normalized potentiation and depression (G_#_/G_1_) for (**a**) P 1.5%, (**b**) P 6%, and (**c**) P 10.5% concentrations. (**d**) Schematic of a three-layer fully connected ANN, including the input, hidden, and output layers for recognizing MNIST handwritten digits. (**e**) Simulated recognition rates with varying numbers of hidden neurons. Error bars indicate standard deviations.

Finally, to validate the neuromorphic computing capabilities of the proposed synaptic transistor, we designed a three-layer perceptron network model to simulate the training of MNIST handwritten digits. The developed artificial neural network (ANN) comprises input, hidden, and output layers, as depicted in Figure 9d. The input layer features 784 neurons, each corresponding to the binary MNIST data of 28 × 28 pixels. The output layer consists of 10 neurons, each corresponding to the digits from 0 to 9. The hidden layer, with 200 neurons, facilitates information transfer between the input and output layers, aiding the neural network in learning and processing a number of features and patterns. Each neuron is connected to others in the next layer through synapses, and the strength of these connections, acting as synaptic weights, is related to the normalized potentiation and depression conductance of PSG-based EDL synaptic transistors, as illustrated in Figure 9a–c. The normalized conductance is calculated by dividing each conductance by the minimum conductance (G_#_/G_1_). Potentiation and depression properties in terms of accuracy in learning and recognition simulations are represented by the ratio of maximum to minimum conductance values (G_max_/G_min_), referred to as the dynamic range (DR) of conductance modulation. The DR values increased with the rise in P concentration, reaching 1.47, 2.03, and 3.58, respectively. A higher DR value is expected to improve simulation accuracy, suggesting that the recognition rate is anticipated to rise as the P concentration increases.

(3) $$AR=\frac{MAX\left|G_{p}\left(n\right)-G_{d}\left(n\right)\right|}{G_{p}\left(30\right)-G_{d}\left(30\right)}\;for\;n=1\;to\;30$$

Equation (2) was used to extract the asymmetry ratio (AR) [60]. G_p_(n) and G_d_(n) represent the conductance values of potentiation and depression, respectively, for the nth iteration. The learning accuracy increases as the AR approaches zero and decreases with the increase in P concentration, reaching 0.96/0.52/0.49.


(4) G={(Gmaxα−Gminα)×w+Gminα}1α if α≠0,Gmin×(Gmax/Gmin)w if α=0.

In addition, to assess the linearity of conductance, we calculated the nonlinearity coefficient (α) using Equation (3) [61]. Here, G_max_ and G_min_ represent the maximum and minimum conductance, respectively, while w serves as an internal variable ranging from 0 to 1. The nonlinearity coefficient α governs either potentiation (α_p_) or depression (α_d_), with the ideal value for the nonlinearity factor being 1. In the developed synaptic transistor, the values of α_p_ and α_d_ corresponding to P concentrations are 25, −15 for P 1.5%, 3.59, −2.9 for P 6%, and 2.05, −1.35 for P 10.5%. Notably, the 10.5% device exhibited values of α_p_ and α_d_ closest to the ideal value of 1.

We leveraged normalized conductance and extracted factors to design the synaptic weights of the ANN. Subsequently, we trained the model using 60,000 MNIST training datasets and conducted recognition tests using 10,000 different test datasets. In Figure 9e, the simulation results of recognition rates are presented as the number of neurons in the hidden layer varies from 10 to 300. With 10 hidden neurons, the recognition rates were 14.2 for P 1.5%, 49.58 for P 6%, and 72.27 for P 10.5%. Interestingly, for P 1.5%, the maximum recognition rate remained low at 22.1 even as the number of hidden neurons increased up to 300. The difference in recognition rates between P 6% and 10.5% devices decreased after 50 hidden neurons. Beyond 100 hidden neurons, both devices exhibited an excellent recognition rate, reaching a maximum of 91.33.

## 4. Conclusions

This study investigated PSG-based electrolyte gate synaptic transistors and unveiled promising approaches for emulating diverse synaptic signal strengths, offering a hardware perspective on selective mimicry of human synaptic behaviors. The fabrication of P-doped PSG electrolyte films, achieved through a convenient spin-coating method and varying phosphorus concentrations, provides a platform for systematic exploration. The frequency-dependent (C-*f*) characteristics established a pivotal link between capacitance and phosphorus concentration, underscoring the versatility of the PSG-EDLT platform. The chemical structure analysis of the electrolyte film elucidated the PSG-EDL mechanism, providing crucial insights into the underlying processes. By fabricating PSG-EDLTs with different phosphorus concentrations, our study delved into the intricacies of synaptic behaviors. The double-sweep transfer curves revealed counterclockwise hysteresis windows, whereas the single-spike EPSC, PPF, frequency and pulse-number-dependent EPSC, P/D characterizations successfully emulated biological synaptic behaviors. Remarkably, the synaptic transistors exhibited excellent performance in handwritten MNIST training simulations, achieving high recognition rates that correlated with increasing phosphorus concentration. Therefore, PSG-based EDLTs combined with precise P concentration control present a promising approach for selectively mimicking human synaptic signal strength. This work contributes valuable insights to the field of neuromorphic systems, paving way to developing sophisticated hardware that reflects the complexity of biological synaptic behaviors.

## Data Availability

Data are contained within the article.
